# Trends in the Incidence of Human Papillomavirus-Associated Cancers by County-Level Income and Smoking Prevalence in the United States, 2000-2018

**DOI:** 10.1093/jncics/pkac004

**Published:** 2022-03-03

**Authors:** Yueh-Yun Lin, Haluk Damgacioglu, Ryan Suk, Chi-Fang Wu, Yenan Zhu, Ana P Ortiz, Sehej Kaur Hara, Kalyani Sonawane, Ashish A Deshmukh

**Affiliations:** 1 Center for Health Services Research, Department of Management, Policy, and Community Health, UTHealth School of Public Health, Houston, TX, USA; 2 Department of Biostatistics and Epidemiology, Graduate School of Public Health, Medical Sciences Campus, University of Puerto Rico, San Juan, Puerto Rico, USA; 3 General Outbreak & Jail Team (GOJ), COVID-19 Contact Tracing & Contact Monitoring Unit (CTCMU), Houston Health Department City of Houston, Houston, TX, USA; 4 Center for Healthcare Data, Department of Management, Policy and Community Health, School of Public Health, UTHealth Science Center at Houston, Houston, TX, USA

## Abstract

Human papillomavirus (HPV)-associated cancer burden is rising in the United States. Trends in the incidence by county-level income and smoking prevalence remain undescribed. We used the Surveillance, Epidemiology, and End Results 21 database to ascertain HPV-associated cancers during 2000-2018. Trends were estimated by county-level income and smoking prevalence quartiles. Anal and vulvar cancer incidence among women and anal cancer incidence among men increased markedly in the lowest-income counties, whereas the increases were slower in the highest-income counties (eg, for vulvar cancer, incidence increased 1.9% per year, 95% confidence interval [CI] = 0.9% to 2.9%, in the lowest-income counties vs 0.8% per year, 95% CI = 0.6% to 1.1%, in the highest-income counties). In recent years, cervical cancer incidence plateaued (0.0% per year [95% CI = −0.5% to 0.5%]) in the highest-income counties; in the lowest-income counties, the annual percentage change was 1.6% per year (95% CI = −0.7% to 4.0%). Counties with high smoking prevalence had marked increases in incidence compared with their counterparts (eg, anal cancer among men increased 4.4% per year [95% CI = 2.7% to 6.0%] for those living in counties with the highest smoking prevalence vs 1.2% per year [95% CI = 0.7% to 1.7%] for those living in counties with the lowest smoking prevalence). Improved and targeted prevention is needed to combat the widening disparities.

In the era of collective decline in cancer incidence, human papillomavirus (HPV)-associated anal, oropharyngeal, and vulvar cancer incidence is rising rapidly, while cervical cancer incidence has stabilized in recent years in the United States (1-3). Risk factors for HPV-associated cancers (eg, current smoking, risky sexual behaviors) (4-7) are more prevalent in poorer counties (8-11). Therefore, trends in HPV-associated cancer incidence in low-income counties and those with high smoking prevalence might differ from their counterparts but remain undescribed. Understanding calendar trends in the incidence of HPV-associated cancers by these county-level attributes can help unravel disparities and inform targeted prevention interventions.

We analyzed the Surveillance, Epidemiology, and End Results 21 (SEER-21) dataset. HPV-associated cancers were identified based on the International Classification of Diseases for Oncology-3 site and histology codes (Supplementary Table 1, available online). We ascertained median household income from the 2011-2015 Census American Community Survey and county-level current smoking prevalence from the National Cancer Institute’s Small Area Estimates for Cancer-Related Measures database (12,13). We categorized these attributes into quartiles based on the population distribution across counties. We used SEER*Stat version 8.3.9 to estimate incidence rates per 100* *000 person-years (age adjusted to the 2000 US standard population). We quantified calendar trends in incidence and calculated the annual percentage changes (APCs) and the average annual percentage changes (AAPCs) using the National Cancer Institute’s Joinpoint Regression Analysis program (version 4.9.0). To determine whether the trends were different from zero, a *t* test was used when there was no joinpoint and a z-test was used when there were one or more joinpoints. Statistical significance was assessed at an α level of *P* < .05, and all hypotheses were 2-sided (14,15).

During 2000-2018, 252* *648 HPV-associated cancers were identified. The incidence of HPV-associated cancers collectively and for individual types was higher in the lowest household income counties and those with the highest level of smoking prevalence when compared with their counterparts (Supplementary Table 2, available online).

Among women living in the lowest-income counties, although not statistically significant, cervical cancer incidence increased 1.6% per year (95% confidence interval [CI] = −0.7% to 4.0%) in recent years (2011-2018) after a significant decline of 2.8% per year (95% CI = −3.8% to −1.7%) during 2000-2011 (Figure 1, A;Supplementary Table 3, available online). In the highest-income counties, after an initial decline (APC_2000__-__2003_ = −3.9%, 95% CI = −5.5% to −2.3%), cervical cancer incidence stabilized in recent years (APC_2003__-__2008_ = −0.6%, 95% CI = −1.7% to 0.5%; APC_2008__-__2011_ = −3.2%, 95% CI = −6.5% to −1.9%; APC_2011__-__2018_ = 0.0%, 95% CI = −0.5% to 0.5%). AAPCs for oropharyngeal cancer in the lowest- and highest-income counties were 1.3% (95% CI = −0.3% to 2.8%) and 0.1% (95% CI = −0.3% to 0.5%), respectively. Anal (3.2% per year, 95% CI = 1.8% to 4.7%) and vulvar (1.9% per year, 95% CI = 0.9% to 2.9%) cancer incidence increased rapidly in the lowest-income counties; the increases were relatively slower in the highest-income counties (AAPC_anal_ = 2.6%, 95% CI = 2.0% to 3.3%; AAPC_vulvar_ = 0.8%, 95% CI =  0.6% to 1.1%).

**Figure 1.**
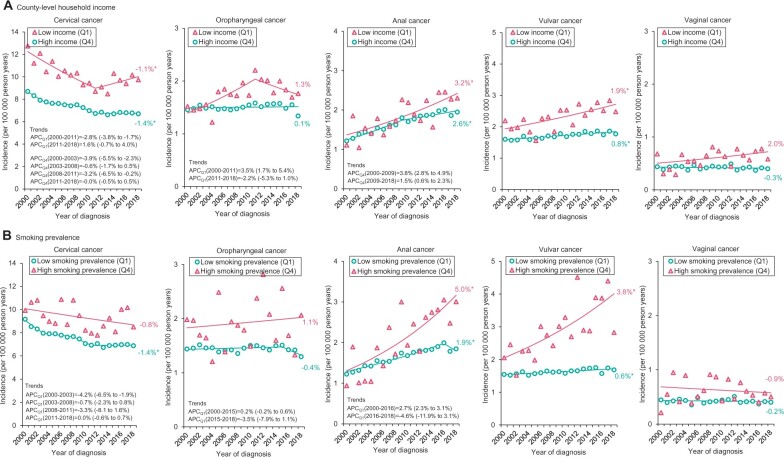
Incidence trends of human papillomavirus (HPV)-associated cancers according to county-level household income and current smoking prevalence among women: Surveillance, Epidemiology, and End Results 21 (SEER-21; 2000-2018). **A**) Trends in incidence rates (per 100* *000 person-years) in HPV-associated cancers by the highest- and lowest-income quartiles. **B**) Trends in incidence rates (per 100* *000 person-years) in HPV-associated cancers by the highest- and lowest smoking prevalence quartiles. Values given beside the annual percentage changes (APCs) within parentheses are the 95% confidence intervals. County-level income quartile values for men and women: Q1 = $9330 to 29* *640, Q4 = $39* *410 to $82* *930; county-level smoking prevalence for men: Q1 = 9.1% to 22.1%, Q4 = 29.3% to 44.7%; county-level smoking prevalence for women: Q1 = 2.9% to 18.3, Q4 = 26.6% to 53.2%. *Statistically significant at *P* < .05 and all hypotheses were 2-sided. A *t* test was used when there was no joinpoint, and a z-test was used when there were one or more joinpoints to determine the statistical significance of trends.

For counties with high smoking prevalence, the AAPC for cervical cancer was −0.8% (95% CI = −1.7% to 0.1%). In counties with low smoking prevalence, cervical cancer incidence stabilized in recent years (APC_2011__-__2018_ = 0.0%, 95% CI = −0.6% to 0.7%) after initial decreases (Figure 1, B;Supplementary Table 3, available online). Anal (AAPC = 5.0%, 95% CI = 2.9% to 7.2%) and vulvar (AAPC = 3.8%, 95% CI = 2.1% to 5.6%) cancer incidence increased markedly in counties with high smoking prevalence, whereas the increases were relatively slower in counties with low smoking prevalence (AAPC_anal_ = 1.9%, 95% CI = 1.0% to 2.7%; AAPC_vulvar_ = 0.6%, 95% CI = 0.3% to 0.9%). No statistically significant change in trends occurred for vaginal cancer incidence by county-level income and smoking prevalence.

Among men, from 2000 to 2018, oropharyngeal cancer incidence increased at 2.1% per year (95% CI = 1.2% to 2.9%) in the lowest-income counties and at 1.7% per year (95% CI = 1.0% to 2.5%) in the highest-income counties (Figure 2, A;Supplementary Table 3, available online). Anal cancer incidence increased rapidly in the lowest-income counties (AAPC = 3.9%, 95% CI = 2.8% to 5.1%) but relatively slowly in the highest-income counties (AAPC = 1.5%, 95% CI  =  0.9% to 2.0%).

**Figure 2.**
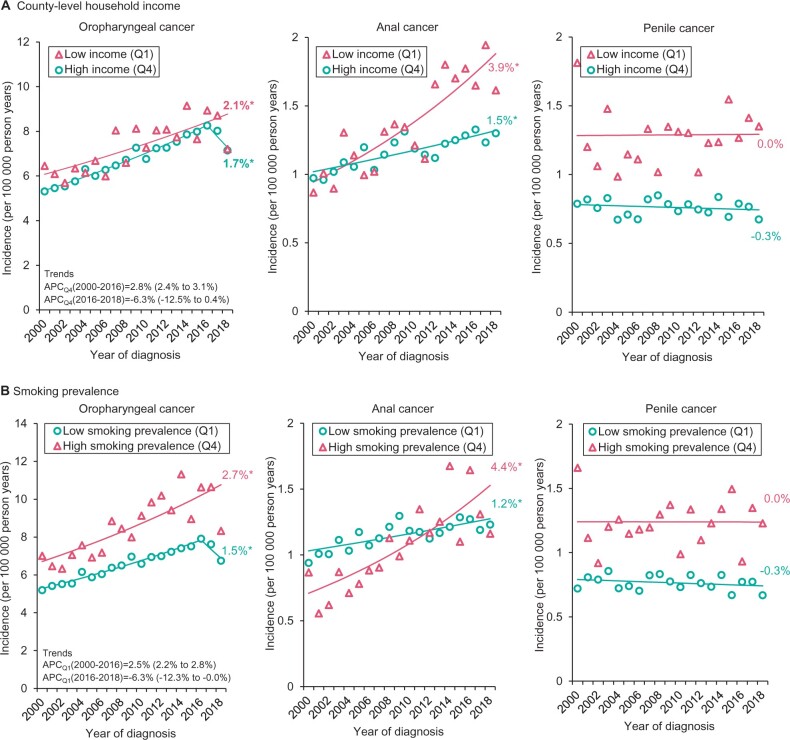
Incidence trends of human papillomavirus (HPV)-associated cancers according to county-level household income and current smoking prevalence among men: Surveillance, Epidemiology, and End Results 21 (SEER-21; 2000-2018). **A**) Trends in incidence rates (per 100* *000 person-years) in HPV-associated cancers by the highest- and lowest-income quartiles. **B**) Trends in incidence rates (per 100* *000 person-years) in HPV-associated cancers by the highest and lowest smoking prevalence quartiles. Values given beside the annual percentage changes (APCs) within parentheses are the 95% confidence intervals. County-level income quartile values for men and women: Q1 = $9330 to 29* *640, Q4 = $39* *410 to $82* *930; county-level smoking prevalence for men: Q1 = 9.1% to 22.1%, Q4 = 29.3% to 44.7%; county-level smoking prevalence for women: Q1 = 2.9% to 18.3%, Q4 = 26.6% to 53.2%. *Statistically significant at *P* < .05 and all hypotheses were 2-sided. A *t* test was used when there was no joinpoint, and a z-test was used when there were one or more joinpoints to determine the statistical significance of trends.

For counties with the highest smoking prevalence, male oropharyngeal (AAPC = 2.7%, 95% CI = 1.7% to 3.7%) and anal cancer (AAPC = 4.4%, 95% CI = 2.7% to 6.0%) incidence increased more rapidly compared with counties with low smoking prevalence (AAPC_oropharyngeal_ = 1.5%, 95% CI = 0.8% to 2.2%; AAPC_anal_ = 1.2%, 95% CI = 0.7% to 1.7%) (Figure 2, B;Supplementary Table 3, available online). No statistically significant changes occurred for penile cancer, irrespective of county-level income and smoking prevalence.

During 2000-2018, higher incidence rates of HPV-associated cancers were observed in counties with the lowest household incomes and the highest smoking prevalence. Furthermore, disparities in HPV-associated anal, oropharyngeal, and vulvar cancer incidence grew between low- and high-income counties and those with the highest smoking prevalence compared with their counterparts.

Smoking has been identified as a risk factor for HPV-associated cancers. Although the exact mechanism of action remains unclear, smoking is believed to impair immune function, prohibiting the ability to clear HPV infection (4). Smoking may also inhibit apoptosis, promoting tumor growth (16). Smoking and risky sexual behaviors are highly correlated with poverty (9). Therefore, marked increases in HPV-associated cancers in low-income counties could be attributable to a combined effect of smoking and increased HPV exposure.

Recent stabilization in cervical cancer incidence in high-income counties and the reversal of declining incidence in low-income counties is troubling and needs further investigation (17,18). These trends are consistent with plateauing of cervical cancer incidence observed nationally during a similar calendar duration in a recent study that utilized data from all US states and another study that documented rising cervical cancer incidence in Puerto Rico (US territory, where >40% women live in poverty) (2,19). Marked increases in anal, oropharyngeal, and vulvar cancer incidence combined with the high absolute incidence in low-income counties and those with high smoking prevalence are troubling. Currently, there are no evidence-based screening recommendations for these cancers; therefore, their prevention solely relies on primary prophylaxis through HPV vaccination. Although HPV vaccination coverage remains greater for adolescents below the poverty level, coverage in rural counties that generally have low median household income is 15 percentage points lower than their counterparts (20). If no improvement in HPV vaccination coverage is achieved in low-income counties, these growing disparities will likely worsen in future years (21-23).

The strength of our study is the use of high-quality data on cancer incidence and county-level income and smoking prevalence. Our study’s limitation is that county-level data do not capture within-county variation in income and smoking prevalence and that inferences at the individual level cannot be drawn from our analysis. Furthermore, to ensure data completeness, SEER allows a delay of 22 months; however, reporting of cases diagnosed in an outpatient facility may be delayed. As a result, the trends in most recent years may erroneously appear to have decreased.

In conclusion, declining cervical cancer incidence has started to reverse, and marked increases in anal, oropharyngeal, and vulvar cancer incidence occurred in the disadvantaged counties. Targeted public health interventions are urgently needed to reduce growing disparities.

## Funding

Research reported in this publication was supported by the National Cancer Institute award numbers R01CA232888, U54CA096300, U54CA096297, and the National Institute on Minority Health and Health Disparities award number K01MD016440.

## Notes

**Role of the funders:** The funders had no role in the design of the study; the collection, analysis, and interpretation of the data; the writing of the manuscript; or the decision to submit the manuscript for publication.

**Disclaimer:** The content is solely the responsibility of the authors and does not necessarily represent the official views of the National Cancer Institute and National Institute on Minority Health and Health Disparities.

**Author contributions:** Conceptualization, YL, HD, AAD; Methodology: YL, HD, AAD; Investigation: All authors; Formal analysis: YL, HD; Writing original draft: YL, HD, AAD; Writing, editing, and revision: All authors; Manuscript approval: All authors.

**Disclosures:** Dr Ortiz reported receiving personal fees from serving as a consultant to Merck outside submitted work. The other authors have no conflicts of interest to disclose.

## Data Availability

The Surveillance Epidemiology and End Results (SEER) data are publicly available at https://seer.cancer.gov/data/. The county attributes are available at https://seer.cancer.gov/seerstat/variables/countyattribs/.

## Supplementary Material

pkac004_Supplementary_Data
