# Supplementary material for: Trends in the Incidence of Human Papillomavirus-Associated Cancers by County-Level Income and Smoking Prevalence in the United States, 2000-2018
Source: JNCI Cancer Spectr. 2022 Mar 3;6(2):pkac004. doi: 10.1093/jncics/pkac004 (PMC8891496; doi:10.1093/jncics/pkac004)
Supplement: pkac004_Supplementary_Data [file pkac004_supplementary_data.pdf]

SUPPLEMENTARY MATERIALS

Trends in the Incidence of Human Papillomavirus-Associated Cancers by County-Level Income and Smoking Prevalence in the United States, 2000-2018

Yueh-Yun Lin, MS,*[1] Haluk Damgacioglu, PhD,*[1] Ryan Suk, PhD,[1] Chi-Fang Wu, PhD,[1] Yenan Zhu, MS,[1] Ana P. Ortiz, PhD, MPH,[2] Sehej Kaur Hara, MPH, BDS[3] Kalyani Sonawane, PhD,[1,4] Ashish A. Deshmukh, PhD, MPH[1]

* Authors with equal contribution

[1] Center for Health Services Research, Department of Management, Policy, and Community Health, UTHealth School of Public Health, Houston, Texas

[2] University of Puerto Rico Comprehensive Cancer Center, San Juan, Puerto Rico

[3] City of Houston, Houston Health Department, Houston, Texas

[4] Center for Healthcare Data, Department of Management, Policy and Community Health, School of Public Health, UT Health Science Center at Houston, Houston, Texas

Correspondence

Ashish A. Deshmukh, PhD, MPH

Center for Health Services Research

Department of Management, Policy and Community Health

UTHealth School of Public Health, 1200 Pressler Street, RAS-E 329

Houston, TX

Tel: 713-500-9180

Fax: 713-500-9493

Email: Ashish.A.Deshmukh@uth.tmc.edu

Supplementary Tables

Supplementary Table 1. Case definition: ICD-O-3 and histology codes

Supplementary Table 2. Incidence of HPV-associated cancers (collective and by specific sites) according to county-level income and smoking prevalence: SEER-21 (2000-2018)

Supplementary Table 3. Trends in incidence of HPV-associated cancers incidence rates according to county-level income and smoking prevalence: SEER-21 (2000-2018)

Supplementary Table 1. Case definition: ICD-O-3 and histology codes

Cancer	ICD-O-3 site codes	Histology codes
Anal cancer	C21.0-C21.8	8050–8084, 8120–8131
Cervical cancer	C53.0-C53.9	8010–8671, 8940–8941
Oropharyngeal cancer	C01.9, C02.4, C02.8, C05.1-C05.2, C09.0-C09.1, C09.8-C09.9, C10.0-C10.4, C10.8-C10.9, C14.0, C14.2, C14.8	8050–8084, 8120–8131
Penile cancer	C60.0-C60.9	8050–8084, 8120–8131
Vaginal cancer	C52.9	8050–8084, 8120–8131
Vulvar cancer	C51.0-C51.9	8050–8084, 8120–8131

Supplementary Table 2. Incidence of HPV-associated cancers according to county-level income and smoking prevalence among men and women: SEER-21 (2000-2018)[a,b]

Cancer site	Median household income				Smoking prevalence			
	Q1		Q4		Q1		Q4	
	Case	Rate (95% CI)	Case	Rate (95% CI)	Case	Rate (95% CI)	Case	Rate (95% CI)
Men								
All	5331	10.1 (9.8 to 10.3)	72 177	8.9 (8.8 to 8.9)	65 709	8.6 (8.5 to 8.7)	7410	11.0 (10.7 to 11.2)
Oropharyngeal	4015	7.4 (7.2 to 7.7)	57 270	6.9 (6.9 to 7.0)	51 714	6.7 (6.6 to 6.7)	5958	8.7 (8.5 to 8.9)
Anal	701	1.4 (1.3 to 1.5)	9397	1.2 (1.2 to 1.2)	8725	1.2 (1.1 to 1.2)	690	1.1 (1.0 to 1.2)
Penile	635	1.3 (1.2 to 1.4)	5510	0.8 (0.7 to 0.8)	5270	0.8 (0.7 to 0.8)	762	1.2 (1.1 to 1.3)
Women								
All	9943	17.5 (17.1 to 17.8)	120 244	13.5 (13.4 to 13.6)	111 703	12.6 (12.6 to 12.7)	3834	16.8 (16.3 to 17.4)
Cervical	5480	10.1 (9.9 to 10.4)	61 828	7.2 (7.2 to 7.3)	63 863	7.5 (7.4 to 7.5)	1909	9.3 (8.9 to 9.7)
Oropharyngeal	1076	1.8 (1.7 to 1.9)	14 035	1.5 (1.5 to 1.5)	13 340	1.4 (1.4 to 1.5)	498	1.9 (1.7 to 2.1)
Anal	1133	1.89 (1.8 to 2.0)	15 891	1.7 (1.7 to 1.7)	15 432	1.7 (1.6 to 1.7)	519	2.1 (1.9 to 2.3)
Vulvar	1380	2.3 (2.2 to 2.4)	16 113	1.7 (1.7 to 1.8)	15 154	1.6 (1.6 to 1.7)	748	2.9 (2.7 to 3.1)
Vaginal	366	0.6 (0.5 to 0.7)	3 912	0.4 (0.4 to 0.4)	3914	0.4 (0.4 to 0.4)	160	0.6 (0.5 to 0.7)

[a]County-level income quartile values for men and women: Q1 = $9330 to 29 640, Q4 = $39 410 to $82 930
[b]County-level smoking prevalence for men: Q1 = 9.1% to 22.1%, Q4 = 29.3% to 44.7%; County-level smoking prevalence for women: Q1 = 2.9% to 18.3%), Q4 = 26.6% to 53.2%

Supplementary Table 3. Trends in incidence of HPV-associated cancers incidence rates according to county-level income and smoking prevalence: SEER-21 (2000-2018)[a]

Cancer site	Attribute	Quartile	Period	Trend		Overall	
				APC (95% CI)	*P*[b]	AAPC (95% CI)	*P*[b]
Women							
All	Income	Q1	2000 - 2012	-0.6% (-1.1% to 0.0%)	0.06	-0.2% (-0.5% to 0.8%)	0.63
			2012 - 2018	1.6% (-0.1% to 3.3%)	0.07		
		Q4	2000 - 2002	-2.5% (-6.0% to 1.2%)	0.16	-0.5% (-0.9% to -0.1%)	0.01
			2002 - 2018	-0.2% (-0.4% to -0.1%)	0.002		
	Smoking prevalence	Q1	2000 - 2003	-2.1% (-4.1% to -0.1%)	0.04	-0.6% (-1% to -0.3%)	<0.001
			2003 - 2018	-0.3% (-0.5% to -0.2%)	0.001		
		Q4	2000 - 2018	0.9% (0.3% to 1.4%)	0.005	0.9% (0.3% to 1.4%)	0.005
Cervical	Income	Q1	2000 - 2011	-2.8% (-3.8% to -1.7%)	<0.001	-1.1% (-2.1% to -0.1%)	0.03
			2011 - 2018	1.6% (-0.7% to 4.0%)	0.17		
		Q4	2000 - 2003	-3.9% (-5.5% to -2.3%)	0.001	-1.4% (-2.0% to -0.8%)	<0.001
			2003 - 2008	-0.6% (-1.7% to 0.5%)	0.23		
			2008 - 2011	-3.2% (-6.5% to 0.2%)	0.06		
			2011 - 2018	0.0% (-0.5% to 0.5%)	0.98		
	Smoking prevalence	Q1	2000 - 2003	-4.2% (-6.5% to -1.9%)	0.003	-1.4% (-2.4% to -0.6%)	0.001
			2003 - 2008	-0.7% (-2.3% to 0.8%)	0.28		
			2008 - 2011	-3.3% (-8.1% to 1.6%)	0.16		
			2011 - 2018	0.0% (-0.6% to 0.7%)	0.88		
		Q4	2000 - 2018	-0.8% (-1.7% to 0.1%)	0.07	-0.8% (-1.7% to 0.1%)	0.07
Oropharyngeal	Income	Q1	2000 - 2011	3.5% (1.7% to 5.4%)	0.001	1.3% (-0.3% to 2.8%)	0.11
			2011 - 2018	-2.2% (-5.3% to 1.0%)	0.16		
		Q4	2000 - 2018	0.1% (-0.3% to 0.5%)	0.65	0.1% (-0.3% to 0.5%)	0.65
	Smoking prevalence	Q1	2000 - 2015	0.2% (-0.2% to 0.6%)	0.32	-0.4% (-1.2% to 0.4%)	0.28
			2015 - 2018	-3.5% (-7.9% to 1.1%)	0.12		
		Q4	2000 - 2018	1.1% (-1.2% to 3.5%)	0.34	1.1% (-1.2% to 3.5%)	0.34
Anal	Income	Q1	2000 - 2018	3.2% (1.8% to 4.7%)	<0.001	3.2% (1.8% to 4.7%)	<0.001
		Q4	2000 - 2009	3.8% (2.8% to 4.9%)	<0.001	2.6% (2.0% to 3.3%)	<0.001
			2009 - 2018	1.5% (0.6% to 2.3%)	0.002		

		Smoking prevalence	Q1	2000 - 2016	2.7% (2.3% to 3.1%)	<0.001	1.9% (1.0% to 2.7%)	<0.001
				2016 - 2018	-4.6% (-11.9% to 3.1%)	0.21		
			Q4	2000 - 2018	5.0% (2.9% to 7.2%)	<0.001	5.0% (2.9% to 7.2%)	<0.001
	Vulvar	Income	Q1	2000 - 2018	1.9% (0.9% to 2.9%)	0.001	1.9% (0.9% to 2.9%)	0.001
			Q4	2000 - 2018	0.8% (0.6% to 1.1%)	<0.001	0.8% (0.6% to 1.1%)	<0.001
		Smoking prevalence	Q1	2000 - 2018	0.6% (0.3% to 0.9%)	<0.001	0.6% (0.3% to 0.9%)	<0.001
			Q4	2000 - 2018	3.8% (2.1% to 5.6%)	<0.001	3.8% (2.1% to 5.6%)	<0.001
	Vaginal	Income	Q1	2000 - 2018	2.0% (-0.2% to 4.2%)	0.07	2.0% (-0.2% to 4.2%)	0.07
			Q4	2000 - 2018	-0.3% (-1.0% to 0.4%)	0.42	-0.3% (-1.0% to 0.4%)	0.42
		Smoking prevalence	Q1	2000 - 2018	-0.2% (-1.0% to 0.5%)	0.50	-0.2% (-1% to 0.5%)	0.50
			Q4	2000 - 2018	-0.9% (-4.2% to 2.3%)	0.54	-0.9% (-4.2% to 2.3%)	0.54
Men								
	All	Income	Q1	2000 - 2018	2.1% (1.3% to 2.8%)	<0.001	2.1% (1.3% to 2.8%)	<0.001
			Q4	2000 - 2016	2.4% (2.0% to 2.7%)	<0.001	1.5% (0.7% to 2.2%)	<0.001
				2016 - 2018	-5.4% (-11.8% to 1.4%)	0.11		
		Smoking prevalence	Q1	2000 - 2016	2.1% (1.8% to 2.4%)	<0.001	1.2% (0.5% to 1.9%)	<0.001
				2016 - 2018	-5.7% (-11.6% to 0.6%)	0.07		
			Q4	2000 - 2018	2.5% (1.6% to 3.4%)	<0.001	2.5% (1.6% to 3.4%)	<0.001
	Oropharyngeal	Income	Q1	2000 - 2018	2.1% (1.2% to 2.9%)	<0.001	2.1% (1.2% to 2.9%)	<0.001
			Q4	2000 - 2016	2.8% (2.4% to 3.1%)	<0.001	1.7% (1.0% to 2.5%)	<0.001
				2016 - 2018	-6.3% (-12.5% to 0.4%)	0.06		
		Smoking prevalence	Q1	2000 - 2016	2.5% (2.2% to 2.8%)	<0.001	1.5% (0.8% to 2.2%)	<0.001
				2016 - 2018	-6.3% (-12.3% to 0.0%)	0.05		
			Q4	2000 - 2018	2.7% (1.7% to 3.7%)	<0.001	2.7% (1.7% to 3.7%)	<0.001
	Anal	Income	Q1	2000 - 2018	3.9% (2.8% to 5.1%)	<0.001	3.9% (2.8% to 5.1%)	<0.001
			Q4	2000 - 2018	1.5% (0.9% to 2.0%)	<0.001	1.5% (0.9% to 2.0%)	<0.001
		Smoking prevalence	Q1	2000 - 2018	1.2% (0.7% to 1.7%)	<0.001	1.2% (0.7% to 1.7%)	<0.001
			Q4	2000 - 2018	4.4% (2.7% to 6.0%)	<0.001	4.4% (2.7% to 6.0%)	<0.001

Penile	Income	Q1	2000 - 2018	-0.0% (-1.3% to 1.4%)	0.96	-0.0% (-1.3% to 1.4%)	0.96
		Q4	2000 - 2018	-0.3% (-1.0% to 0.4%)	0.40	-0.3% (-1.0% to 0.4%)	0.40
	Smoking prevalence	Q1	2000 - 2018	-0.3% (-1.0% to 0.3%)	0.26	-0.3% (-1% to 0.3%)	0.26
		Q4	2000 - 2018	0.0% (-1.3% to 1.3%)	0.99	0% (-1.3% to 1.3%)	0.99

[a]County-level income quartile values for men and women: Q1 = $9330 to 29 640, Q4 = $39 410 to $82 930
County-level smoking prevalence for men: Q1 = 9.1% to 22.1%, Q4 = 29.3% to 44.7%; County-level smoking prevalence for women: Q1 = 2.9% to 18.3%), Q4 = 26.6% to 53.2%
[b] t-test was used when there was no joinpoint and a z-test was used when there were one or more joinpoints to determine whether the trends were different from zero. Statistical significance was assessed at an α level of $P < 0.05$, and all hypotheses were 2-sided.
